# Integrating NLP to Enhance Algorithmic Identification of Metastatic and Castration‐Resistant Prostate Cancer in Large Claims‐Based Studies

**DOI:** 10.1002/cam4.71406

**Published:** 2025-12-21

**Authors:** Shannon R. Stock, Joshua A. Parrish, Michael T. Burns, Jessica L. Janes, Justin Waller, Amanda M. De Hoedt, Sameer Ghate, Jeri Kim, Irene M. Shui, Stephen J. Freedland

**Affiliations:** ^1^ Department of Surgery Durham VA Health Care System Durham North Carolina USA; ^2^ Department of Mathematics and Computer Science College of the Holy Cross Worcester Massachusetts USA; ^3^ Merck & Co., Inc. Kenilworth New Jersey USA; ^4^ Department of Urology Cedars‐Sinai Medical Center Los Angeles California USA

**Keywords:** castration‐resistant prostate cancer detection, claims data algorithms, metastatic disease detection, natural language processing

## Abstract

**Purpose:**

Accurate classification of prostate cancer (PC) disease states defined by the presence or absence of metastasis and castration resistance (CRPC) is critical but challenging in population‐based research. As chart review is not feasible on a large scale, accurate automated methods are needed.

**Methods:**

We conducted a retrospective study using data from the Veterans Affairs Health Care System to evaluate algorithms for identifying CRPC and metastatic PC, with manual chart review as the gold standard. Our analysis included 8336 patients for CRPC classification and 721 for metastatic disease classification. For CRPC classification, we assessed one novel algorithm using criteria including rising prostate‐specific antigen levels or progression to metastatic disease while receiving androgen deprivation therapy or initiating CRPC‐specific treatments. For metastatic disease detection, we assessed four algorithms based on: ICD codes alone, natural language processing (NLP) alone, a novel algorithm combining ICD codes and treatment patterns, and an enhanced version of the novel algorithm integrating NLP, evaluating the sensitivity and specificity of each. Positive and negative predictive values were reported across a range of assumed disease prevalence.

**Results:**

Out of 8336 patients with PC, 1190 (14.3%) were identified as having CRPC through chart review, with the CRPC algorithm achieving 85.1% sensitivity and 96.1% specificity. Among 721 patients evaluated for metastatic disease, 179 (24.8%) were identified as having metastatic disease through chart review. The algorithm combining ICD codes, treatment patterns, and NLP demonstrated the highest sensitivity (94.4%) and high specificity (93.0%), while other methods had lower sensitivity with varied specificity.

**Conclusions:**

Our findings suggest that our CRPC algorithm and the combined ICD codes, treatment patterns, and NLP algorithm for metastasis are effective automated approaches for identifying advanced states of PC. In particular, integrating NLP boosted sensitivity for metastatic classification with minimal specificity trade‐off, highlighting the value of a multifaceted approach to large‐scale PC research.

## 1 Purpose

Prostate cancer (PC) is a predominantly indolent disease, with most patients diagnosed with localized cancer [1]. However, for some patients, PC progresses to more advanced states including metastatic disease and develops resistance to androgen deprivation therapy (ADT), a condition known as castration‐resistant PC (CRPC). Nearly all patients who succumb to PC reach the state of metastatic CRPC.

For researchers conducting database studies, accurate classification of metastatic and CRPC disease states is essential. The ability to reliably identify these advanced disease states allows for the robust analysis of treatment patterns, survival outcomes, and healthcare resource utilization. Such analyses are essential for guiding clinical decision‐making and shaping healthcare policies. While manual chart review of electronic health records (EHRs) remains the gold standard for classification of these disease states, manual abstraction on a population‐based scale is rarely feasible due to cohort size and often databases don't allow researchers access to the full EHR (e.g., Medicare) [2, 3]. Consequently, automated methods using administrative data have become desirable for large‐scale research.

Several algorithms have been developed to classify disease states in administrative databases, particularly using International Classification of Diseases (ICD) codes, treatment patterns, and laboratory markers (when available) like rising prostate‐specific antigen (PSA) [4, 5]. These methods are commonly used to infer CRPC. However, structured fields alone often provide incomplete information about treatment timing, laboratory‐confirmed castration status, or the clinical rationale for therapy. ICD‐10 codes for hormone‐sensitive versus castration‐resistant states are inconsistently applied in practice, and medication data may not distinguish treatment indications, leading to misclassification of disease state.

This limitation has prompted the use of proxies such as treatment patterns and PSA dynamics, which may no longer be sufficient given many treatments are now used in earlier disease stages [6, 7, 8]. Finally, in true claims‐only data, laboratory markers are not available.

Similarly, metastatic disease is often classified using ICD codes and treatment patterns. In recent years, natural language processing (NLP) has emerged as a complementary tool, offering the potential to extract detailed information from unstructured text within EHRs [9, 10]. By combining structured data with NLP, researchers can overcome some of the inherent limitations of structured fields and capture nuanced clinical data that improves the accuracy of algorithmic classification of disease states.

To provide reliable automated methods to classify advanced PC states, we evaluated an algorithm for classifying CRPC that considers ICD codes, treatment patterns, and PSA measurements while patients are on ADT. Second, we evaluated different approaches for classifying metastatic disease based on ICD codes, treatment patterns, and NLP. In both cases, manual chart abstraction was the gold standard for validating the accuracy of these algorithms.

## 2 Methods

We conducted a retrospective study from the Veterans Affairs Health Care System (VAHCS), utilizing data from the VA Corporate Data Warehouse, which contains comprehensive patient information, including billing codes, pharmacy records, free‐text radiology reports, and clinical notes. The study included patients of male sex, aged ≥ 18 who were diagnosed with PC, defined by the presence of two or more ICD‐9 or ICD‐10 codes for PC on separate dates. There were no exclusion criteria specific to this study. We evaluated the performance of one novel algorithm for CRPC vs. chart abstraction among 8336 patients diagnosed between January 1, 2000, and December 31, 2020. To identify metastatic disease, we evaluated 4 algorithms among 721 patients diagnosed between January 1, 2012, and December 31, 2016. Both the CRPC and metastatic cohorts included patients whose chart abstraction data were available as part of other existing studies, providing a robust dataset to validate and test our algorithms against manual abstraction.

### 2.1 Manual Chart Abstraction

To validate algorithmic classifications, manual chart abstraction was performed following a standardized protocol developed for prior VA PC studies [5, 11]. A detailed abstraction manual was developed before data collection, specifying variable definitions, decision rules, and adjudication procedures. Trained abstractors then reviewed the full EHR, including clinical notes, pathology and radiology reports, laboratory values, and medication histories, to confirm PC diagnosis, classify disease state, and verify inclusion criteria. To assess inter‐rater reliability, 10% of charts were randomly selected for independent re‐review by a secondary abstractor. Discrepancies were resolved by consensus, with final adjudication by the principal investigator. This process enabled capture of detailed clinical information (e.g., timing of CRPC onset, treatment start dates) not available in structured data.

For CRPC, chart abstraction confirmed castration by bilateral orchiectomy, continuous ADT, or testosterone < 50 ng/dL. Patients were classified as CRPC during castration by either PSA rise (≥ 2 ng/mL and ≥ 1.25× nadir) or radiographic progression. The CRPC date was the earliest occurrence of either event. For metastasis, chart abstraction involved review of radiology reports without considering treatment history. Each scan was categorized as positive (confirmed metastasis) or negative (no or uncertain metastasis).

### 2.2 Algorithm to Identify CRPC

**FIGURE 1:**
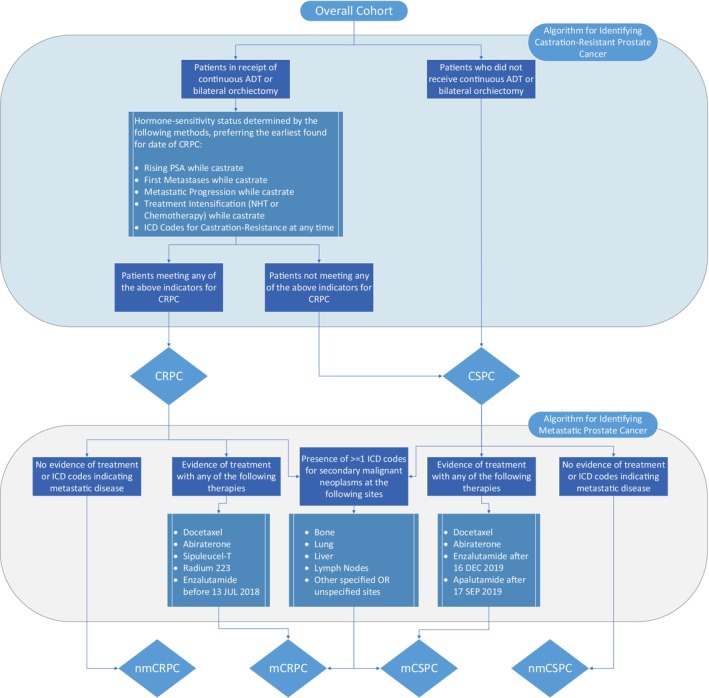
Algorithms for identifying castration‐resistant and metastatic prostate cancer.

The CRPC algorithm identified patients undergoing medical or surgical castration, defined as either continuous ADT or a bilateral orchiectomy. Patients meeting castration criteria were classified as CRPC based on ICD‐10 code Z19.2 (specific to CRPC), rising PSA, metastatic progression, or initiation of CRPC‐specific therapy (Figure 1). The earliest qualifying date defined CRPC onset.

### 2.3 Algorithms to Identify Metastatic PC

We evaluated four algorithms for detecting metastatic disease. The first used ICD‐10 codes for secondary malignant neoplasms. The second employed NLP to scan free‐text radiology reports for evidence of metastases, which we previously reported and demonstrated to be highly accurate [11]. The third was a novel algorithm combining ICD‐10 codes for metastases with administration of treatments such as Abiraterone, Enzalutamide, Apalutamide, Docetaxel, Sipuleucel‐T, or Radium‐223, restricted to time periods when these drugs were approved only for metastatic PC (Figure 1). The fourth algorithm augmented the ICD codes and treatment approach with our NLP model.

### 2.4 Statistical Analysis

Sensitivity (true positive rate) and specificity (true negative rate) were calculated for each algorithm using chart abstraction as the gold standard. Positive predictive value (PPV) and negative predictive value (NPV) were estimated across a range of potential metastatic PC and CRPC prevalence values using calculated sensitivities and specificities.

## 3 Results

Descriptive statistics of the CRPC and metastasis cohorts are provided in eTables S1 and S2, respectively. Sensitivity, specificity, and predictive value estimates (PPV and NPV, assuming 5%–50% prevalence) for all algorithms are presented in Table 1. Among 8336 patients used to evaluate the CRPC algorithm, 1190 (14.3%) were classified as CRPC by chart review. Sensitivity and specificity (95% CI) for the CRPC algorithm were 85.1% (83.0–87.1) and 96.1% (95.6–96.5), respectively, indicating a strong ability to distinguish true CRPC while minimizing false positives.

**TABLE 1:** Summary of the diagnostic ability of different classification methods.

Measure	Prevalence	Algorithm to identify CRPC	Algorithms to identify metastasis			
		Structured data alone	ICD alone	NLP alone	ICD ± treatment	ICD ± treatment + NLP
		Value (95% CI)	Value (95% CI)	Value (95% CI)	Value (95% CI)	Value (95% CI)
Sensitivity		**85.13 (82.98, 87.10)**	**67.60 (60.21, 74.39)**	**53.07 (45.48, 60.56)**	**89.39 (83.92, 93.49)**	**94.41 (89.97, 97.29)**
Specificity		**96.05 (95.58, 96.49)**	**96.31 (94.36, 97.73)**	**97.42 (95.70, 98.58)**	**95.02 (92.83, 96.69)**	**92.99 (90.50, 94.99)**
Positive predictive value (PPV)	5%	53.17 (50.25, 56.06)	49.09 (38.26, 60.00)	51.96 (38.77, 64.87)	48.57 (39.45, 57.78)	41.48 (34.23, 49.11)
	10%	70.56 (68.08, 72.93)	67.06 (56.68, 76.00)	69.54 (57.21, 79.58)	66.60 (57.90, 74.29)	59.94 (52.36, 67.08)
	15%	79.20 (77.21, 81.06)	76.37 (67.51, 83.41)	78.38 (67.98, 86.09)	76.00 (68.60, 82.11)	70.38 (63.57, 76.39)
	20%	84.36 (82.75, 85.84)	82.08 (74.64, 87.69)	83.70 (75.05, 89.77)	81.77 (75.58, 86.67)	77.10 (71.20, 82.09)
	25%	87.79 (86.48, 88.99)	85.93 (79.70, 90.48)	87.26 (80.04, 92.12)	85.68 (80.49, 89.66)	81.78 (76.73, 85.94)
	30%	90.24 (89.16, 91.22)	88.70 (83.46, 92.43)	89.80 (83.76, 93.76)	88.49 (84.14, 91.77)	85.23 (80.91, 88.71)
	35%	92.07 (91.18, 92.88)	90.80 (86.38, 93.88)	91.71 (86.63, 94.97)	90.62 (86.96, 93.33)	87.88 (84.19, 90.80)
	40%	93.50 (92.75, 94.17)	92.43 (88.70, 95.00)	93.20 (88.92, 95.90)	92.29 (89.19, 94.55)	89.98 (86.83, 92.44)
	45%	94.64 (94.01, 95.20)	93.75 (90.60, 95.89)	94.39 (90.78, 96.63)	93.62 (91.01, 95.51)	91.68 (89.00, 93.75)
	50%	95.57 (95.05, 96.04)	94.82 (92.17, 96.61)	95.36 (92.33, 97.23)	94.72 (92.53, 96.30)	93.09 (90.82, 94.83)
Negative predictive value (NPV)	5%	99.19 (99.07, 99.29)	98.26 (97.86, 98.59)	97.53 (97.12, 97.88)	99.42 (99.11, 99.62)	99.68 (99.43, 99.83)
	10%	98.31 (98.07, 98.52)	96.40 (95.58, 97.07)	94.92 (94.11, 95.62)	98.77 (98.14, 99.20)	99.34 (98.80, 99.64)
	15%	97.34 (96.96, 97.67)	94.40 (93.16, 95.42)	92.17 (90.96, 93.22)	98.07 (97.07, 98.73)	98.95 (98.10, 99.42)
	20%	96.27 (95.75, 96.73)	92.24 (90.58, 93.63)	89.25 (87.66, 90.66)	97.28 (95.90, 98.21)	98.52 (97.33, 99.18)
	25%	95.09 (94.42, 95.69)	89.92 (87.82, 91.68)	86.16 (84.19, 87.93)	96.41 (94.61, 97.62)	98.04 (96.47, 98.92)
	30%	93.78 (92.93, 94.52)	87.40 (84.87, 89.56)	82.89 (80.55, 84.99)	95.43 (93.17, 96.97)	97.49 (95.51, 98.61)
	35%	92.30 (91.28, 93.22)	84.66 (81.70, 87.22)	79.40 (76.73, 81.84)	94.33 (91.57, 96.22)	96.87 (94.42, 98.26)
	40%	90.64 (89.42, 91.73)	81.68 (78.29, 84.65)	75.69 (72.70, 78.45)	93.07 (89.77, 95.36)	96.15 (93.18, 97.85)
	45%	88.76 (87.32, 90.04)	78.41 (74.61, 81.79)	71.73 (68.45, 74.79)	91.63 (87.73, 94.36)	95.31 (91.76, 97.38)
	50%	86.59 (84.93, 88.09)	74.83 (70.62, 78.61)	67.49 (63.97, 70.82)	89.95 (85.40, 93.20)	94.33 (90.11, 96.82)

Among the 721 patients used to evaluate the algorithms for metastasis, 179 (24.8%) were classified as having metastatic disease by chart review. Across algorithms for metastasis, sensitivity improved with the inclusion of additional data sources. The ICD + treatment + NLP algorithm performed best, demonstrating the highest sensitivity (94.4%) and high specificity (93.0%), followed by the ICD + treatment algorithm (89.4% and 95.0%). Algorithms using ICD or NLP alone showed lower sensitivity but excellent specificity (> 96%).

## 4 Conclusions

Real‐world EHR and claims database studies have become increasingly important for population‐level research. However, performing detailed chart reviews for every patient in such studies is generally infeasible. To address the need for identifying patients with CRPC and/or metastatic disease, several algorithms have been developed [4, 5, 9]. These algorithms demonstrate the utility of structured data, such as ICD codes, laboratory results, and treatment information, although the added value of integrating NLP remains underexplored.

To address this gap, we evaluated our CRPC algorithm, which relies solely on structured data readily available in claims databases, and observed high sensitivity (85.1%) and specificity (96.1%). By comparison, Candelieri‐Surette et al. incorporated NLP into their approach, achieving sensitivity of 97.9% and specificity of 99.2% [4]. Similarly, our novel algorithm for identifying metastatic PC, which integrates ICD codes, treatment patterns, and NLP, improved sensitivity (94.4%) with minimal impact on specificity (93.0%) compared to using any individual method alone. This result reflects the complementary strengths of these approaches, where each method demonstrates high specificity (all > 90% individually) but variable sensitivity, leading to missed cases. By combining these methods, the integrated algorithm achieved substantial gains in sensitivity while maintaining high specificity. These findings demonstrate the value of augmenting structured‐data‐based algorithms with NLP to enhance the classification of metastatic PC.

Importantly, for situations like claims databases where NLP is unavailable, the algorithms based on structured data such as our CRPC algorithm and our ICD plus treatment algorithm for metastatic PC (sensitivity 89.4%, specificity 95.0%) also performed well. This underscores their utility in resource‐limited contexts.

This study has several limitations. First, it spans a time during which treatment patterns evolved, which may impact the generalizability of our findings. Future studies should account for these changes to ensure applicability of the algorithms in contemporary settings. Additionally, our algorithms rely on EHR data, limiting their use in datasets lacking EHR access. Finally, further investigation is needed to validate these algorithms in studies conducted both inside and outside the VAHCS.

These findings highlight the importance of using integrated approaches for accurately classifying advanced PC states in large‐scale studies. By combining multimodal data and harnessing the capabilities of NLP, sensitivity and specificity are improved compared to prior methods, providing a valuable framework for conducting population‐level studies.

## Author Contributions

**Shannon R. Stock:** conceptualization, methodology, formal analysis; writing – original draft; writing – review editing; supervision; data curation. **Joshua A. Parrish:** writing review and editing; project administration; data curation; supervision. **Michael T. Burns:** data curation; supervision; project administration; writing – review and editing. **Jessica L. Janes:** formal analysis; data curation; writing – review and editing. **Justin Waller:** project administration; supervision; data curation; writing – review and editing. **Amanda M. De Hoedt:** supervision; project administration; Writing – review and editing. **Sameer Ghate:** writing – review and editing; project administration. **Jeri Kim:** writing – review and editing; project administration. **Irene M. Shui:** conceptualization; writing – review and editing; project administration; supervision. **Stephen J. Freedland:** conceptualization; funding acquisition; Writing – review and editing; project administration; supervision.

## Funding

This work was supported by Merck Sharp & Dohme LLC, a subsidiary of Merck & Co Inc.

## Ethics Statement

This study followed Strengthening the Reporting of Observational Studies in Epidemiology (STROBE) reporting guidelines with institutional review board approval from the Durham VHA. Data were collected with waivers of informed consent in accordance with 45 CFR §46.

## Conflicts of Interest

The authors declare no conflicts of interest.

## Supporting information


**eTable S1:** Descriptive statistics of the study cohort used to evaluate castration‐resistant prostate cancer algorithms.
**eTable S2:** Descriptive statistics of the study cohort used to evaluate metastatic prostate cancer algorithms.

## Data Availability

The data use PHI and cannot be released.
