# Supplementary material for: Integrating NLP to Enhance Algorithmic Identification of Metastatic and Castration‐Resistant Prostate Cancer in Large Claims‐Based Studies
Source: Cancer Med. 2025 Dec 21;14(24):e71406. doi: 10.1002/cam4.71406 (PMC12719047; doi:10.1002/cam4.71406)
Supplement: Supplementary file 1 — eTable S1: Descriptive statistics of the study cohort used to evaluate castration‐resistant prostate cancer algorithms. eTable S2: Descriptive statistics of the study cohort used to evaluate metastatic prostate cancer algorithms. [file CAM4-14-e71406-s001.docx]

**Supplemental Materials**

eTable 1. Descriptive statistics of the study cohort used to evaluate castration-resistant prostate cancer algorithms.

| Variable | Total (N=8336) |
| --- | --- |
| Age at PC Diagnosis |  |
| Median | 63.0 |
| Q1, Q3 | 58.0, 68.0 |
| Missing | 0 |
| Race/Ethnicity |  |
| White, non-Hispanic | 4336 (54.4%) |
| Black, non-Hispanic | 2303 (28.9%) |
| Other, non-Hispanic | 318 (4.0%) |
| Hispanic | 1020 (12.8%) |
| Missing | 359 |
| US Census Region at PC Diagnosis |  |
| Midwest | 2561 (30.7%) |
| Northeast | 982 (11.8%) |
| South | 2301 (27.6%) |
| West | 1737 (20.8%) |
| Puerto Rico | 755 (9.1%) |
| Total Gleason Score at PC Diagnosis |  |
| ≤ 6 | 789 (24.9%) |
| 7 (3 + 4) | 1046 (33.1%) |
| 7 (4 + 3) | 574 (18.1%) |
| 8 | 399 (12.6%) |
| 9-10 | 355 (11.2%) |
| Missing | 5173 |
| PSA at PC Diagnosis |  |
| Median | 6.8 |
| Q1, Q3 | 4.9, 12.0 |
| Missing | 2764 |
| *PC=Prostate Cancer, PSA=Prostate-specific antigen | |

eTable 2. Descriptive statistics of the study cohort used to evaluate metastatic prostate cancer algorithms.

| Variable |  | Total (N=721) |
| --- | --- | --- |
| Age at PC Diagnosis |  |  |
| Median |  | 67 |
| Q1, Q3 |  | 63, 76 |
| Missing |  | 0 |
| Race/Ethnicity |  |  |
| White, non-Hispanic |  | 410 (59.4%) |
| Black, non-Hispanic |  | 216 (31.3%) |
| Other, non-Hispanic |  | 21 (3.0%) |
| Hispanic |  | 43 (6.2%) |
| Missing |  | 31 |
| US Census Region at PC Diagnosis |  |  |
| Midwest |  | 310 (43.0%) |
| Northeast |  | 231 (32.0%) |
| South |  | 105 (14.6%) |
| West |  | 57 (7.9%) |
| Puerto Rico |  | 18 (2.5%) |
| Total Gleason Score at PC Diagnosis |  |  |
| ≤ 6 |  | 78 (18.1%) |
| 7 (3 + 4) |  | 94 (21.9%) |
| 7 (4 + 3) |  | 73 (17.0%) |
| 8 |  | 75 (17.4%) |
| 9-10 |  | 110 (25.6%) |
| Missing |  | 291 |
| PSA at PC Diagnosis |  |  |
| Median |  | 23.0 |
| Q1, Q3 |  | 10.0, 50.9 |
| Missing |  | 227 |
| Evidence of Metastasis(es) |  |  |
| By chart review |  | 179 (24.8%) |
| By ICD codes |  | 141 (19.6%) |
| By Treatment Algorithm |  | 150 (20.8%) |
| By NLP |  | 109 (15.1%) |

*PC = Prostate Cancer, PSA = Prostate-specific antigen
